# Active-sensing-based decentralized control of autonomous mobile agents for quick and smooth collision avoidance

**DOI:** 10.3389/frobt.2022.992716

**Published:** 2022-11-11

**Authors:** Takeshi Kano, Takeru Kanno, Taishi Mikami, Akio Ishiguro

**Affiliations:** ^1^ Research Institute of Electrical Communication, Tohoku University, Sendai, Japan; ^2^ Graduate School of Engineering, Tohoku University, Sendai, Japan

**Keywords:** collision avoidance, active sensing, decentralized control, mobile agents, autonomous systems

## Abstract

There is an increasing demand for multi-agent systems in which each mobile agent, such as a robot in a warehouse or a flying drone, moves toward its destination while avoiding other agents. Although several control schemes for collision avoidance have been proposed, they cannot achieve quick and safe movement with minimal acceleration and deceleration. To address this, we developed a decentralized control scheme that involves modifying the social force model, a model of pedestrian dynamics, and successfully realized quick, smooth, and safe movement. However, each agent had to observe many nearby agents and predict their future motion; that is, unnecessary sensing and calculations were required for each agent. In this study, we addressed this issue by introducing active sensing. In this control scheme, an index referred to as the “collision risk level” is defined, and the observation range of each agent is actively controlled on this basis. Through simulations, we demonstrated that the proposed control scheme works reasonably while reducing unnecessary sensing and calculations.

## 1 Introduction

Recently, labor shortages in the transportation industry have become a serious problem because of declining birth rates and an aging population. Although the transportation industry is an important economic sector, workers experience physical fatigue caused by long working hours. Moreover, recent developments in information and communication technology have increased the workload. Therefore, the development of control schemes for autonomous mobile systems such as automated transport robots in logistics warehouses and flying drones is necessary. In these systems, each mobile agent must reach its destination while avoiding other agents. Thus, safe collision avoidance must be considered in the design of control schemes. In addition to safety, other factors can further improve the utility of these systems. Specifically, if the speed of each agent is maintained at a maximum with minimal acceleration and deceleration, that is, if quick and smooth movement is achieved, agents can reach their destinations quickly with low energy consumption.

Multi-robot systems have been studied from various aspects, such as formation control (Ali et al., 2018), collective transport (Jurt et al, 2022), and task allocation (Valentini et al., 2022). Collision avoidance in multi-robot systems has also been extensively studied, and several methods such as path planning using centralized (Solovey et al., 2016; Yu and LaValle, 2016; Tang et al., 2018) and decentralized (Bekris et al., 2012; Zhou et al., 2017) control, learning (Arai et al., 1997; Godoy et al., 2016; Chen et al., 2017; Long et al., 2018; Fan et al., 2020; Liang et al., 2020), and approaches based on velocity obstacles (Fiorini and Shiller, 1998; Snape et al., 2009, Snape et al., 2010; van der Berg et al., 2011a; van der Berg et al., 2011b; Hennes et al., 2012; Snape et al., 2014; Alonso-Mora et al., 2013; Claes and Tuyls, 2018) have been proposed. However, in these studies, the focus was primarily on avoiding collisions, and the quickness of the agents’ movement was not considered. Conversely, several studies have been conducted to achieve multi-objective tasks. For example, genetic algorithms (Nazarahari et al., 2019; Yang et al., 2019), swarm intelligence algorithms (Hidalgo-Paniagua et al., 2015, Hidalgo-Paniagua et al., 2017), the electrostatic field approach (Bayat et al., 2018), and path planning (Ahmed and Deb, 2011; Ravankar et al., 2016) have been used to achieve multiple objectives, such as reducing the path length and increasing the safety and smoothness of trajectories. Zhu et al. (Zhu et al., 2020) developed a control scheme for multi-robot collision avoidance using a vertical-ellipse-based velocity obstacle method integrated with a dynamic window approach (Fox et al., 1997). Using mobile robots, they demonstrated that multiple objectives (i.e., reducing time consumption and path length and increasing safety) can be achieved with suitable parameters. However, their study aimed to achieve quick movement and a smooth trajectory, and reducing the acceleration and deceleration of the agents was beyond its scope.

**FIGURE 1:**
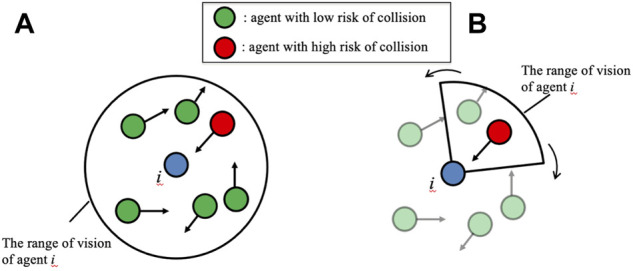
Basic concept of **(A)** our previous control scheme and **(B)** the control scheme proposed in this study. **(A)** In our previous scheme, future motions of agents within a circular range of vision are predicted regardless of the risk of collision (both red and green agents). **(B)** In the control scheme proposed in this study, the range of vision is fan-shaped, and its direction is actively changed. Future motions of agents with a high risk of collision (red agent) are predicted, whereas those of the other agents (transparent green agents) are ignored.

To address this issue, we previously developed a simple decentralized control scheme for multiple mobile agents by modifying the social force model (Helbing and Molnár, 1995), which is a model of pedestrian dynamics, such that the prediction of collisions between mobile agents can be considered (Kano et al., 2021). Through simulations, we demonstrated that this control scheme enables safe collision avoidance while maintaining speed and reducing acceleration and deceleration. However, each agent still had to predict the motion of all agents within the observation range, regardless of the risk of collision (Figure 1A). Thus, unnecessary sensing and calculation were required, and this scheme was unreasonable considering hardware implementation.

This paper proposes a control scheme for mobile agents capable of enabling quick, smooth, and safe movement with limited sensing capability for each agent. For this purpose, we drew inspiration from the active sensing of living organisms, such as the echo location of bats (Yamada et al., 2020). Active sensing is an effective way to reduce the waste associated with sensing costs because important sensory information is actively brought into focus and useless information is neglected. We developed a simple control scheme for the active sensing and motion of each agent (Figure 1B). Subsequently, we investigated whether the proposed control scheme worked effectively through simulations. Moreover, we quantitatively evaluated the speed, smoothness, and safety and compared the proposed control scheme with our previous scheme (Kano et al., 2021).

The remainder of this paper is organized as follows. In Section 2, we briefly review our previous model (Kano et al., 2021) and propose a mathematical model incorporating active sensing. In Section 3, we present several simulation results and demonstrate that the proposed control scheme works satisfactorily. Finally, the conclusions and future work are presented in Section 4.

## 2 Problem statement

We assume that *N* agents are on a two-dimensional (2D) plane with a periodic boundary condition. The position of the agent *i* is denoted by **r**_*i*_. Although the unit vector **e**_*i*_, which represents the direction in which agent *i* intends to move, can be varied temporally in a real scenario, it was kept constant in this study for simplicity. The direction of **e**_*i*_ is determined randomly, that is, each agent aims to move in a different direction. The target speed *v*_0_, that is, the speed in the absence of other agents, is common to all agents. Here, we do not consider the cases in which obstacles exist in the plane.

Our objective was to realize collision avoidance while maintaining speed and smoothness with low sensing and calculation costs. Specifically, we propose a decentralized control scheme for each mobile agent in which the following indices are kept as small as possible while reducing sensing and calculation costs:

$$E_{1}=1-\frac{\int_{0}^{T}\overset{N}{\underset{i=1}{\sum }}\left({\dot{r}}_{i}\cdot e_{i}\right)}{v_{0}NT},$$ (1)

$$E_{2}=\frac{\int_{0}^{T}\overset{N}{\underset{i=1}{\sum }}|{\ddot{r}}_{i}{|}^{2}}{nNT},$$ (2)

$$E_{3}=\frac{\int_{0}^{T}\overset{N}{\underset{i=1}{\sum }}|f_{ij}^{\text{phys}}|}{nNT},$$ (3)

where *T* denotes the total time of the simulation experiment and $f_{ij}^{\text{phys}}$ denotes the physical force vector when agents *i* and *j* collide (Eq. 15). Indices *E*_1_, *E*_2_, and *E*_3_ are small when the agents move quickly, smoothly, and safely, respectively.

In our previous study (Kano et al., 2021), we succeeded in realizing quick, smooth, and safe movement. In other words, we succeeded in reducing the aforementioned indices relative to those obtained with previously proposed models (Helbing and Molnár, 1995; Zanlungo et al., 2011). However, unnecessary sensing and calculations were required in our previous scheme. Herein, we propose a decentralized control scheme that uses active sensing, which is an effective way to reduce the waste associated with sensing costs. Because the sensing cost is reduced, performance deterioration (*i.e.,*, an increase in *E*_1_, *E*_2_, and *E*_3_), is inevitable. However, we attempted to reduce this deterioration to the maximum possible extent.

## 3 Model

In this section, we briefly review our previous model (Kano et al., 2021) (Section 3.1) and propose a mathematical model that incorporates active sensing (Section 3.2).

### 3.1 Outline of our previous model (Kano et al., 2021)

**FIGURE 2:**
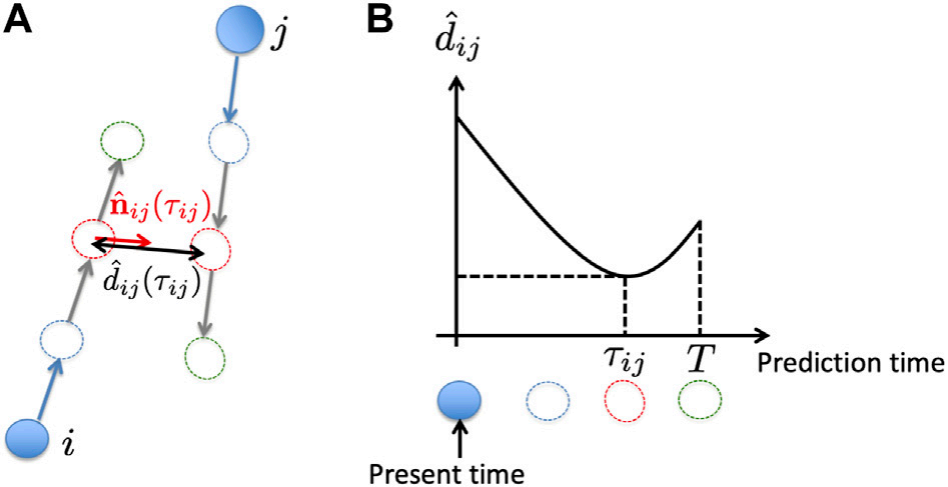
Schematic of the previous model (Kano et al., 2021). **(A)** Based on the present position and velocity of the cars (blue filled circles and arrows, respectively), their future trajectories are estimated by numerically solving a differential equation [Step (ii)] (blue, red, and green dashed circles). **(B)** Schematic of the time evolution of the distance between cars *i* and *j* for the situation shown in **(A)**.

We previously modified the social force model (Helbing and Molnár, 1995), a model of pedestrian dynamics, and proposed a mathematical model of mobile agents, in which each agent was controlled such that it could move quickly and smoothly while avoiding other agents (Kano et al., 2021). In the proposed model, the social force term in the social force model, which originates from psychological effects, was changed to the control input to mobile agents, and it was designed according to the following steps:

Step (i): Each agent detects the relative position and velocity of agents within a distance *R* from itself at every constant time interval.
Step (ii): Each agent predicts the future motion of nearby agents for duration *T* by numerically solving a differential equation in which the social force term is omitted from the social force model, with the initial position and velocity of the agents set to be the same as those obtained in Step (i) (Figure 2).
Step (iii): Based on the numerical calculation performed in Step (ii), the expected time until the distance between agents *i* and *j* has a minimum value or until they collide, *τ*_*ij*_, is derived (Figure 2). Furthermore, the expected distance between agents *i* and *j* after the time interval *τ*_*ij*_, $\hat{d}\left(\tau_{ij}\right)$ , is derived.
Step (iv): The control input is calculated using the values of *τ*_*ij*_ and $\hat{d}\left(\tau_{ij}\right)$ obtained in Step (iii). The equation that determines the control input is derived by modifying the social force term in the social force model (Helbing and Molnár, 1995). It consists of the exclusive volume effect and the avoidance force based on the prediction of future motion, the latter of which increases as *τ*_*ij*_ and $\hat{d}\left(\tau_{ij}\right)$ decrease.

In our previous study (Kano et al., 2021), we demonstrated through simulations that agents could move quickly and smoothly while avoiding each other. However, the calculation cost for each agent is high; each agent must calculate the future motion of all agents within a distance *R* from itself by solving a differential equation [Step (ii)]. A reduction in the calculation cost is required to consider the hardware implementation.

### 3.2 Proposed model

Herein, we propose a mathematical model that incorporates a decentralized control scheme for autonomous mobile agents to achieve quick, smooth, and safe movement with low sensing and calculation costs. For this purpose, an index referred to as the “collision risk level” is introduced for each pair of agents, and each agent is controlled such that it ignores agents whose collision risk level is low. The observation range of each agent is constrained to a fan shape, and its direction is actively controlled to focus on agents whose collision risk is high (Figure 1). Active sensing was used to identify high-risk agents. Furthermore, instead of solving the differential equation in Step (ii) of the previous model, the focused agents were predicted to move at a constant velocity; thus, each agent does not need to know the directions in which other nearby agents intend to move.

Each agent has a circular shape with radius *r* and can move omnidirectionally on a 2D plane. Agents have their own destinations, and their task is to reach these destinations without losing velocity or smoothness, while avoiding other agents. It is assumed that each agent can detect the relative position and velocity of other agents within its fan-shaped observation range.

**FIGURE 3:**
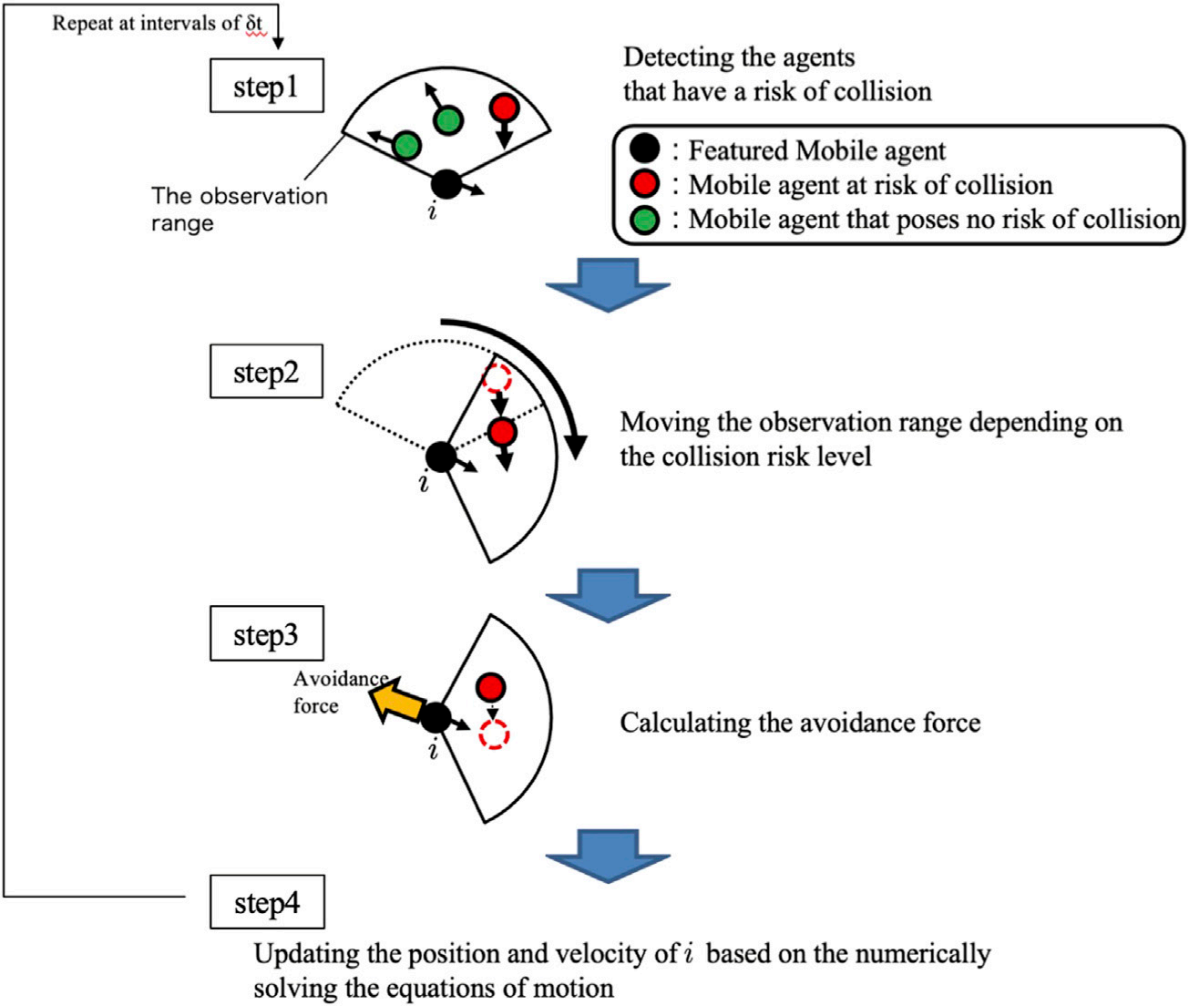
Flow of the algorithm for the proposed control.

The control algorithm for each agent consists of the following four steps (Figure 3).

Step 1: An agent detects other agents within its observation range and calculates the collision risk level.
Step 2: The agent controls the direction of the fan-shaped observation range to focus on agents whose collision risk is high, that is, it performs active sensing.
Step 3: The agent predicts the future motion of agents whose collision risk is high and calculates the avoidance force.
Step 4: The agent moves based on the calculated avoidance force.

In the following section, each step is described in detail.

#### 3.2.1 Calculation of collision risk level (Step 1)

**FIGURE 4:**
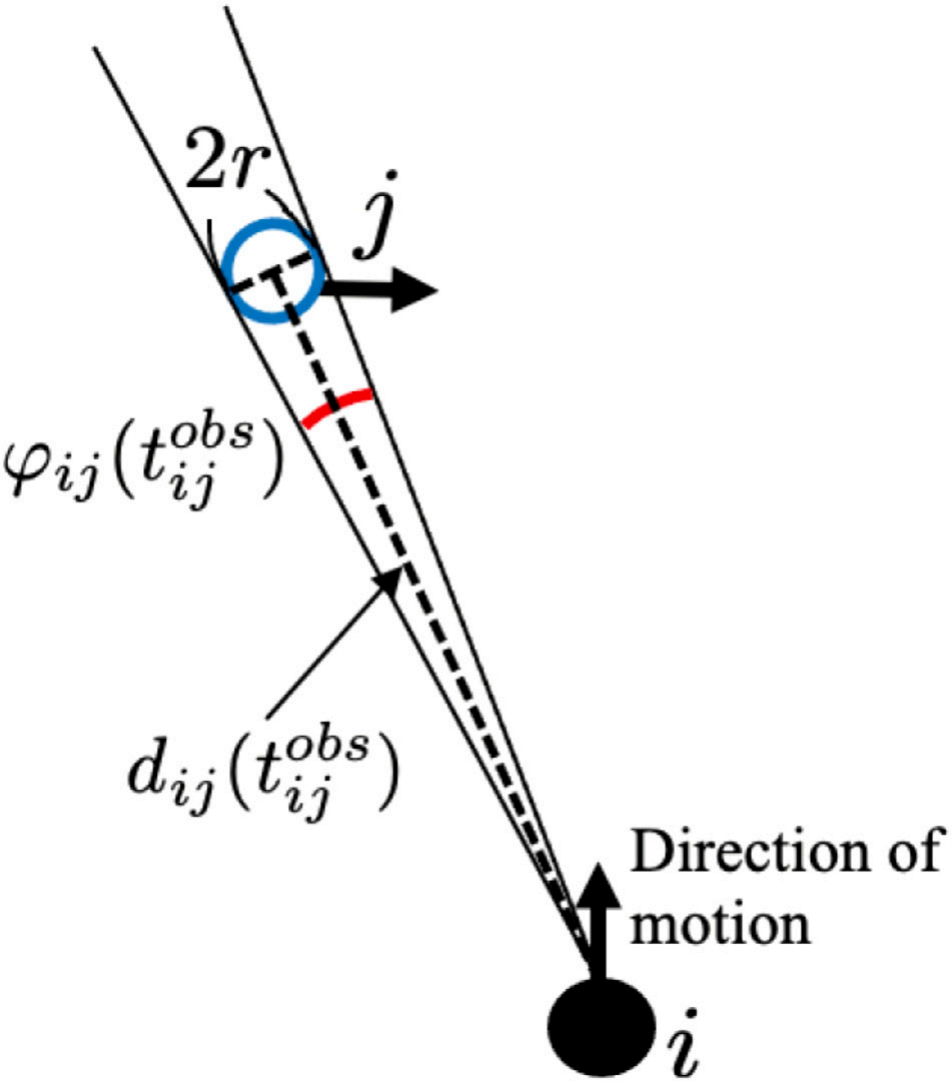
Definitions of $d_{ij}\left(t_{ij}^{\mathrm{obs}}\right)$ and $\varphi_{ij}\left(t_{ij}^{\mathrm{obs}}\right)$ . Here, $t_{ij}^{\mathrm{obs}}$ is the time when agent *j* is detected by agent *i* for the latest time.

**FIGURE 5:**
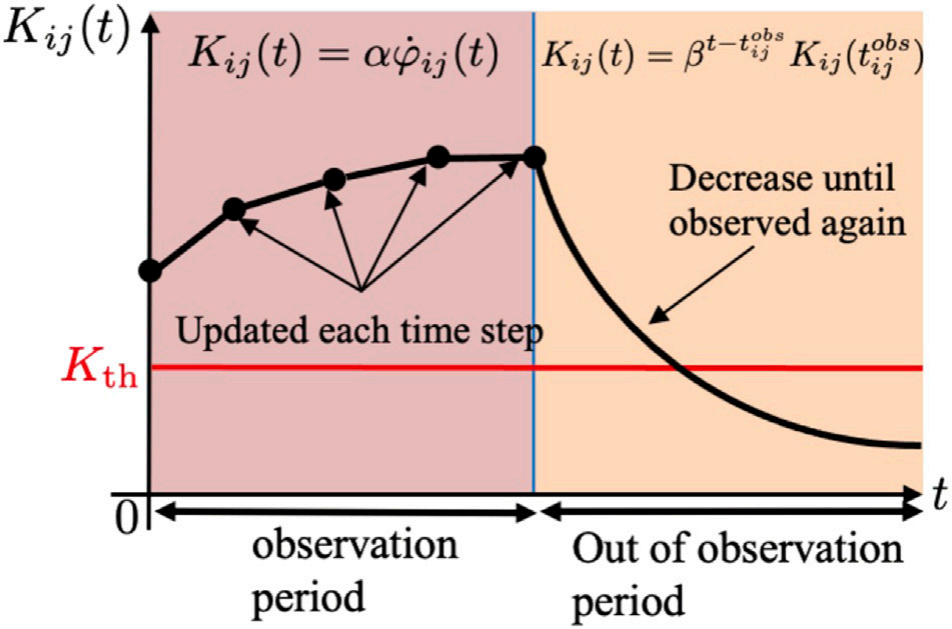
Time evolution of collision risk level *K*_*ij*_.

For every time interval, agent *i* calculates the collision risk level between agents *i* and *j*, defined as *K*_*ij*_, according to the following equation:

$$K_{ij}\left(t\right)=\left\{\begin{array}{cc} \alpha {\dot{\varphi }}_{ij}\left(t_{ij}^{\mathrm{obs}}\right)\; & \left(t=t_{ij}^{\mathrm{obs}}\right),\\ \beta^{t-t_{ij}^{\mathrm{obs}}}K_{ij}\left(t_{ij}^{\mathrm{obs}}\right)\; & \left(t>t_{ij}^{\mathrm{obs}}\right), \end{array}\right.$$ (4)

$$\varphi_{ij}\left(t_{ij}^{\mathrm{obs}}\right)=2\arctan\frac{r}{d_{ij}\left(t_{ij}^{\mathrm{obs}}\right)},$$ (5)

where *α* is a positive constant, *β* is a constant that satisfies 0 < *β* < 1, $t_{ij}^{\mathrm{obs}}$ is the latest time when agent *i* observes agent *j*, and *φ*_*ij*_ is the range of vision of agent *j* as viewed from agent *i* (Figure 4). The meaning of Eq. 4 is as follows: when agent *j* is within the observation range of agent *i*, the collision risk level *K*_*ij*_ is defined as the change rate of the range of vision ${\dot{\varphi }}_{ij}$ . Thus, *K*_*ij*_ increases when agent *j* approaches agent *i*. When agent *j* is outside the observation range of agent *i*, it is difficult to determine the extent to which agent *j* approaches agent *i*. Therefore, we assume that *K*_*ij*_ decreases exponentially (Figure 5). When *K*_*ij*_ is above the threshold *K*_*th*_, agent *i* recognizes that agent *j* has collision risk.

#### 3.2.2 Determination of the observation range (Step 2)

**FIGURE 6:**
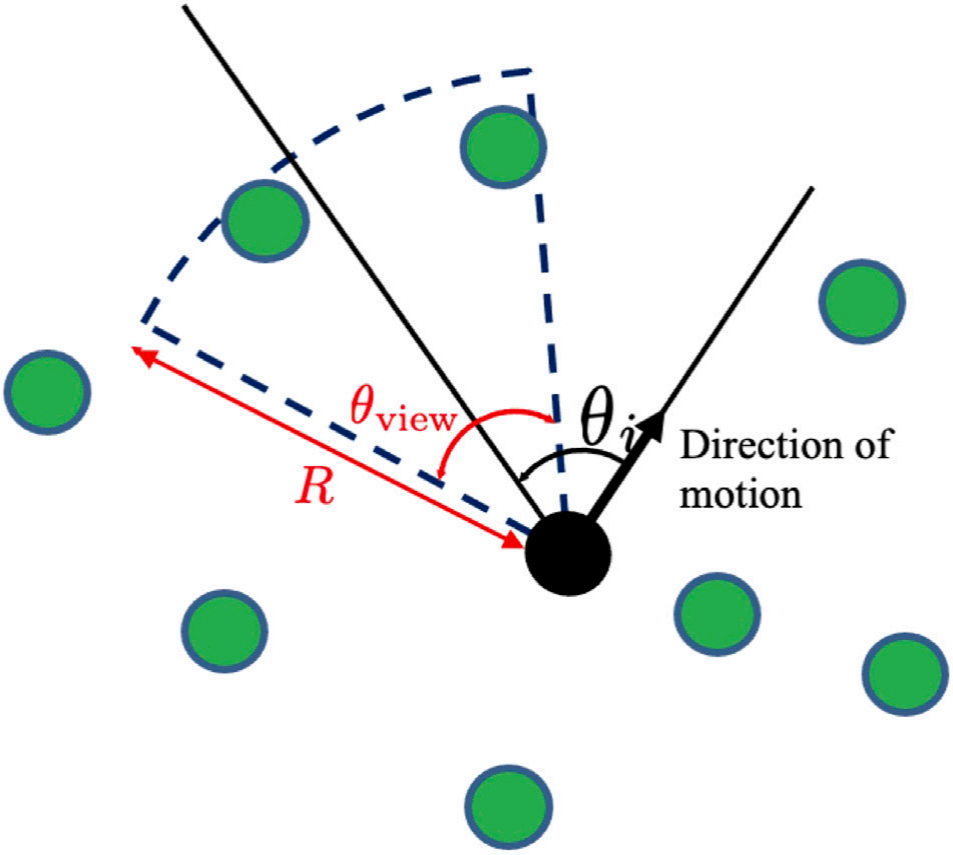
Observation range and definitions of *R*, *θ*_view_, and *θ*_*i*_.

The direction of the fan-shaped observation range with respect to the direction of the movement of agent *i* is defined as *θ*_*i*_ ( − *π* ≤ *θ*_*i*_ < *π*) (Figure 6). Angle *θ*_*i*_ is updated at every time interval Δ*t*_view_ based on a probabilistic distribution function *p*(*θ*_*i*_, *t*), which is given by.

$$f\left(\theta_{i},t\right)=\varepsilon -\gamma |\theta_{i}-\theta_{i,goal}|+\underset{j\in K_{ij}\geq K_{th}}{\sum }K_{ij}\left(t\right)\max\left\{\left(w_{1}-w_{2}|\theta_{i}-{\tilde{\theta }}_{ij}\left(t\right)|\right),0\right\},$$ (6)

$$p\left(\theta_{i},t\right)=\frac{f\left(\theta_{i},t\right)}{\int_{-\pi }^{\pi }f\left(\theta_{i},t\right)d\theta_{i}},$$ (7)

where *ɛ*, *γ*, *w*_1_, and *w*_2_ are positive constants and *θ*_*i*,*goal*_ denotes the direction of the destination with respect to the moving direction of agent *i*. ${\tilde{\theta }}_{ij}\left(t\right)$ denotes the estimated direction of the location of agent *j* with respect to the direction of movement of agent *i*, which is described as

$${\tilde{\theta }}_{ij}\left(t\right)=\arccos\frac{{\dot{r}}_{i}\left(t\right)\cdot {\tilde{r}}_{ij}\left(t\right)}{\left|{\dot{r}}_{i}\left(t\right)\|{\tilde{r}}_{ij}\left(t\right)\right|},$$ (8)

where **r**_*i*_ denotes the position of agent *i* and ${\tilde{r}}_{ij}\left(t\right)$ is the estimated relative position vector of agent *j* with respect to agent *i* at time *t*. The derivation of ${\tilde{r}}_{ij}\left(t\right)$ is given by Eq. 9.

**FIGURE 7:**
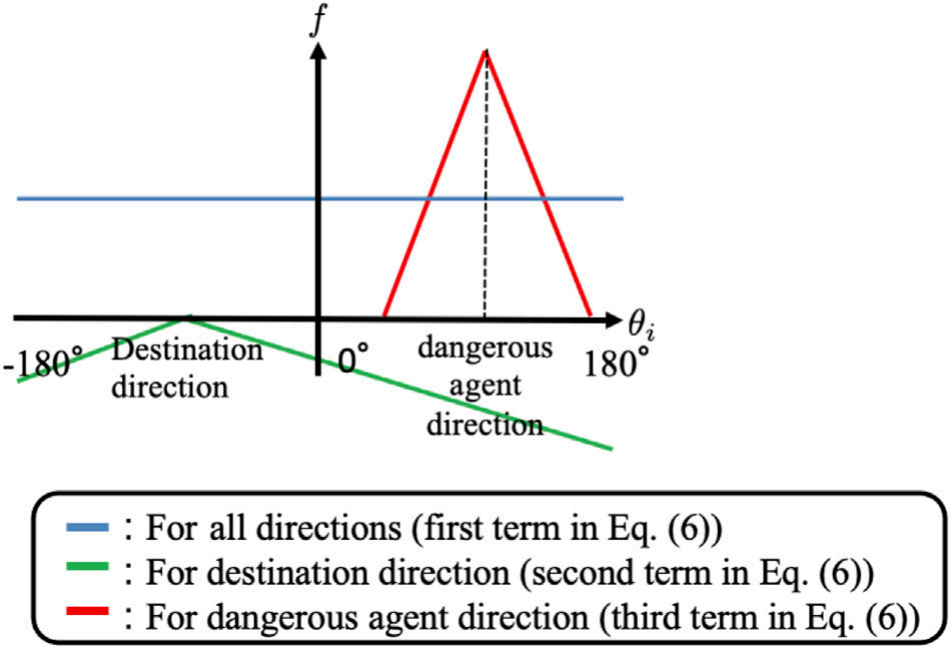
Probability distribution of the direction of the observation range.

Here, *p*(*θ*_*i*_, *t*) is the normalized function of *f*(*θ*_*i*_, *t*) and *f*(*θ*_*i*_, *t*) consists of three terms (Figure 7). The first term on the right side of Eq. 6 indicates that agent *i* pays equal attention in all directions. The second term indicates that agent *i* pays more attention to the destination direction. The third term indicates that agent *i* focuses on agents with collision risk levels. Here, the max function is introduced such that the probability distribution is selectively increased in the direction of agents whose collision risk levels are high. Thus, active sensing focuses on agents expected to collide in the near future.

#### 3.2.3 Calculation of avoidance force (Step 3)

**FIGURE 8:**
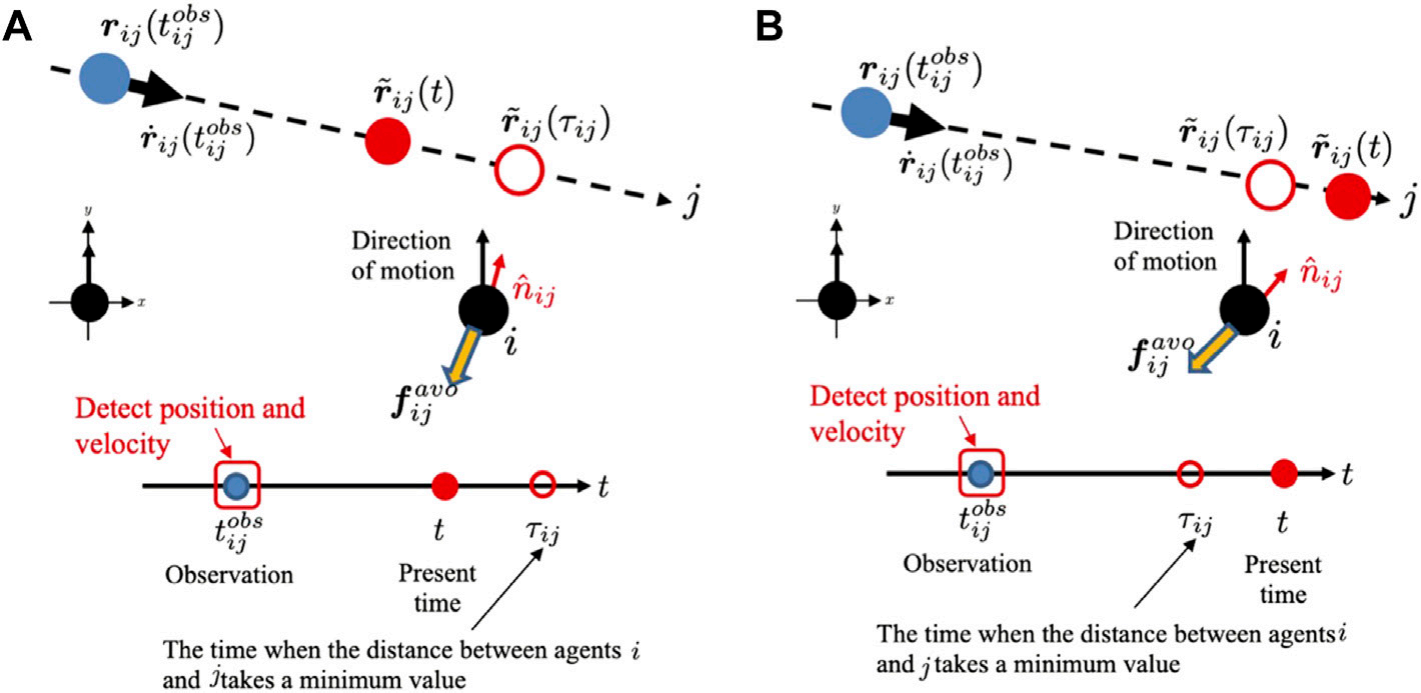
**(A)** Prediction of the future position of mobile agents that have a risk of collision in the case where *t* ≤ *τ*_*ij*_.**(B)** Prediction of the future position of mobile agents that have a risk of collision in the case where *t* > *τ*_*ij*_.

The avoidance force of the agent is calculated for agents whose collision risk level is above threshold *K*_*th*_. We explain how agent *i* generates an avoidance force from agent *j* when *K*_*ij*_ ≥ *K*_*th*_. The basic idea is that the avoidance force vector of agent *i* is generated in the direction opposite to that of agent *j* when agent *j* approaches the closest point (Figure 8A), and that its magnitude is proportional to *K*_*ij*_. The closest point was estimated by assuming that agent *j* moved at a constant velocity after it was observed by agent *i*. However, when agent *j* has already passed through the closest point, it is expected to move away from agent *i* in the near future. Therefore, in this case, the avoidance force vector of agent *i* is generated in the direction opposite to the current estimated position of agent *j* (Figure 8B).

This concept is mathematically described as follows:

$${\tilde{r}}_{ij}\left(t\right)={\dot{r}}_{ij}\left(t_{ij}^{\mathrm{obs}}\right)\left(t-t_{ij}^{\mathrm{obs}}\right)+r_{ij}\left(t_{ij}^{\mathrm{obs}}\right),$$ (9)

$${\tilde{r}}_{ij}\left(\tau_{ij}\right)\cdot {\dot{r}}_{ij}\left(t_{ij}^{\mathrm{obs}}\right)=0,$$ (10)

$${\tilde{r}}_{ij}\left(\tau_{ij}\right)={\dot{r}}_{ij}\left(t_{ij}^{\mathrm{obs}}\right)\left(\tau_{ij}-t_{ij}^{\mathrm{obs}}\right)+r_{ij}\left(t_{ij}^{\mathrm{obs}}\right),$$ (11)

where $r_{ij}\left(t_{ij}^{\mathrm{obs}}\right)$ is the relative position vector of agent *j* with respect to agent *i* when agent *i* observes agent *j* and ${\tilde{r}}_{ij}\left(t\right)$ is the estimated relative position vector of agent *j* with respect to agent *i* at time *t*. Time *τ*_*ij*_ denotes the time at which agent *j* is expected to reach the closest point.

The avoidance force vector $f_{ij}^{\text{avo}}\left(t\right)$ is given by

$$f_{ij}^{\text{avo}}\left(t\right)=-CK_{ij}\left(t\right){\hat{n}}_{ij}\left(t\right),$$ (12)

where *C* is a positive constant and the unit vector ${\hat{n}}_{ij}\left(t\right)$ is defined as

$${\hat{n}}_{ij}\left(t\right)=\left\{\begin{array}{cc} \frac{{\tilde{r}}_{ij}\left(\tau_{ij}\right)}{\left|{\tilde{r}}_{ij}\left(\tau_{ij}\right)\right|}\; & \left(t\leq \tau_{ij}\right),\\ \frac{{\tilde{r}}_{ij}\left(t\right)}{\left|{\tilde{r}}_{ij}\left(t\right)\right|}\; & \left(t>\tau_{ij}\right). \end{array}\right.$$ (13)

Here, agent *j* approaches agent *i* when *t* ≤ *τ*_*ij*_ (Figure 8A) while agent *j* has already passed the closest point and moves away from agent *i* in the case of *t* > *τ*_*ij*_ (Figure 8B).

#### 3.2.4 Equation of motion (Step 4)

By modifying the social force model (Helbing and Molnár, 1995) and using the avoidance force derived in Step 3, the equation of motion for agent *i* can be expressed as

$$m{\ddot{r}}_{i}=a\left(v_{0}e_{i}-{\dot{r}}_{i}\right)+\underset{j}{\sum }f_{ij}^{\text{phy}}+\underset{j\in K_{ij}\geq K_{th}}{\sum }f_{ij}^{\text{soc}},$$ (14)

where *m* denotes the mass of the agent; **e**_*i*_ denotes a unit vector representing the direction in which agent *i* intends to move; *v*_0_ denotes the target speed; and *a* denotes a positive constant. The first term on the right side indicates that the velocity of the agent approaches the desired velocity *v*_0_ **e**_*i*_. The second term denotes the physical interaction between the agents when they make contact with each other, and is described in the same manner as the social force model (Helbing and Molnár, 1995) as

$$f_{ij}^{\text{phys}}=-p\theta \left(2r-d_{ij}\right)n_{ij}+q\theta \left(2r-d_{ij}\right)\left\{\left({\dot{x}}_{j}-{\dot{x}}_{i}\right)\cdot t_{ij}\right\}t_{ij},$$ (15)

where *p* and *q* are positive constants, **n**_*ij*_ = (**r**_*j*_ − **r**_*i*_)/|**r**_*j*_ − **r**_*i*_|, **t**_*ij*_ is a unit vector perpendicular to **n**_*ij*_, *d*_*ij*_ = |**r**_*j*_ − **r**_*i*_|, and *θ*(*x*) = *x* for *x* > 0 and *θ*(*x*) = 0 for *x* ≤ 0.

The third term on the right side of Eq. 14 denotes the control input to the agent and $f_{ij}^{\text{soc}}$ is expressed as

$$f_{ij}^{\text{soc}}=-A\exp\left(-\frac{{\tilde{d}}_{ij}-2r}{B}\right){\tilde{n}}_{ij}+f_{ij}^{\text{avo}},$$ (16)

where *A* and *B* are positive constants, ${\tilde{d}}_{ij}=|{\tilde{r}}_{j}-r_{i}|$ , and ${\tilde{n}}_{ij}=\left({\tilde{r}}_{j}-r_{i}\right)/|{\tilde{r}}_{j}-r_{i}|$ . The first term on the right side of Eq. 16 originates from the social force term in the social force model (Helbing and Molnár, 1995), and represents the repulsive force exerted by agent *j*. However, when agent *j* was not within the observation range, a repulsive force was generated using the estimated relative position (Eq. 9). The second term on the right-hand side denotes the avoidance force derived in Step 3.

It should be noted that the third term on the right side of Eq. 14 uses only the sensory information of agents whose collision risk level is above threshold *K*_*th*_. Thus, agents with a collision risk below *K*_*th*_ are ignored. Moreover, the avoidance force $f_{ij}^{\text{avo}}$ is derived by simply assuming that agent *j* is expected to move at a constant velocity after it is finally observed by agent *i* (Section 3.2.3). The future motion of a nearby agent is calculated by solving the differential equation in our previous model [Step (ii) in Section 3.1]. Thus, the calculation cost for each agent in the proposed model is significantly lower than that in the previous model (Kano et al., 2021).

## 4 Simulation

Simulations were conducted using the settings described in Section 2 to evaluate the performance of the proposed control scheme. The differential equations are discretized with time step *δt*. Because it was difficult to evaluate all the parameters, we first explored the parameters that enabled quick, smooth, and safe movement by hand tuning. We then changed several parameters that would affect the performance, whereas the other parameters were fixed. Unless otherwise noted, the parameter values listed in Table 1 are used.

**TABLE 1:** Parameter values used in the simulation.

Variable	Dimension	Value
*N*	-	20
*L*	m	100
*r*	m	2.00
*a*	kg・s^−1^	6.67 × 10^2^
*m*	kg	1.00 × 10^2^
*v*_0_	m・s^−1^	13.3
*p*	kg・s^−2^	2.22 × 10^4^
*q*	kg・m^−1^・s^−1^	1.67 × 10^2^
*A*	kg・m・s^−2^	8.00 × 10^3^
*B*	m	6.00
*C*	kg・m・s^−2^	1.00 × 10^6^
*R*	m	40.0
*θ*_view_	-	*π*/2
Δ*t*_view_	s	0.15
*α*	s	4.00 × 10^–3^
*β*	-	0.9999
*γ*	-	3.00 × 10^–3^
*ɛ*	-	1.00 × 10^–2^
*w*_1_	-	50.0
*w*_2_	-	60.0
*K*_*th*_	-	6.00 × 10^–4^
*δt*	s	0.0015
*n*	-	2 × 10^4^

The performance indices *E*_1_, *E*_2_, and *E*_3_ were calculated by discretizing Eqs. 1–3:

$$E_{1}=1-\frac{\overset{n}{\underset{k=1}{\sum }}\overset{N}{\underset{i=1}{\sum }}\left(v_{i}\left(k\right)\cdot e_{i}\right)}{v_{0}nN},$$ (17)

$$E_{2}=\frac{\overset{n}{\underset{k=1}{\sum }}\overset{N}{\underset{i=1}{\sum }}|a_{i}{\left(k\right)}^{2}|}{nN},$$ (18)

$$E_{3}=\frac{\overset{n}{\underset{k=1}{\sum }}\overset{N}{\underset{i=1}{\sum }}|f_{ij}^{\text{phys}}\left(k\right)|}{nN},$$ (19)

where *k* denotes the time step, *n* the maximum time step, and *N* the number of agents. The velocities **v**_*i*_(*k*) and accelerations **a**_*i*_(*k*) are given by **v**_*i*_(*k*) = (**r**_*i*_(*k* + 1) − **r**_*i*_(*k*))/*δt* and **a**_*i*_(*k*) = (**v**_*i*_(*k* + 1) − **v**_*i*_(*k*))/*δt*, respectively.

In Section 4.1, we demonstrate that the proposed model performs reasonably well when the parameter values are appropriate. In Section 4.2, we change several key parameters for active sensing and quantitatively evaluate their dependencies to understand the properties of the proposed model. In Section 4.3, the performance of the proposed model is quantitatively compared with that of the previous model.

### 4.1 Representative results

**FIGURE 9:**
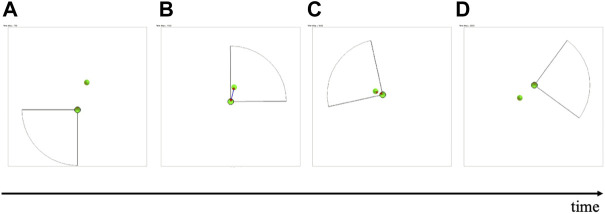
Snapshot of the simulation when *N* = 2. The fan-shaped observation range is shown for one of the agents. Two agents are connected by a line when *K*_*ij*_ is above the threshold *K*_*th*_. The probability distribution of the observation direction (Eqs. 6,7) is indicated by the color of each agent. Red and green denote the directions where the probability distributions are high and low, respectively.

First, we perform a simulation with *N* = 2. The video and snapshots are shown in Supplementary Movie S1 and Figure 9, respectively. In this section, we focus on one of the agents, and explain how they move. First, the agent in focus did not observe any other agents (Figure 9A). When the other agent entered the observation range, the collision risk level increased, and the agent was recognized as having a high risk of collision. Subsequently, the probability distribution of the center angle of the fan-shaped observation range changed, and the agent tended to focus on the other agent (Figure 9B). Consequently, the agent succeeded in avoiding the other agent, and the collision risk level decreased (Figures 9C, D). This result suggests that the proposed control algorithm performed well.

Next, we perform a simulation using *N* = 20 (Supplementary Movie S2). In this case, each agent found other agents with a high collision risk level and tended to focus on them. Subsequently, each agent successfully avoided the other agents.

### 4.2 Parameter dependence

To understand the properties of the proposed model, we performed simulations by changing the radius *R* and central angle *θ*_view_ of the fan-shaped observation range, time interval for updating the fan-shaped observation range Δ*t*_view_, and threshold of the collision risk level *K*_*th*_. For each simulation, the speed, smoothness, and safety were evaluated quantitatively using *E*_1_, *E*_2_, and *E*_3_, respectively.

**FIGURE 10:**
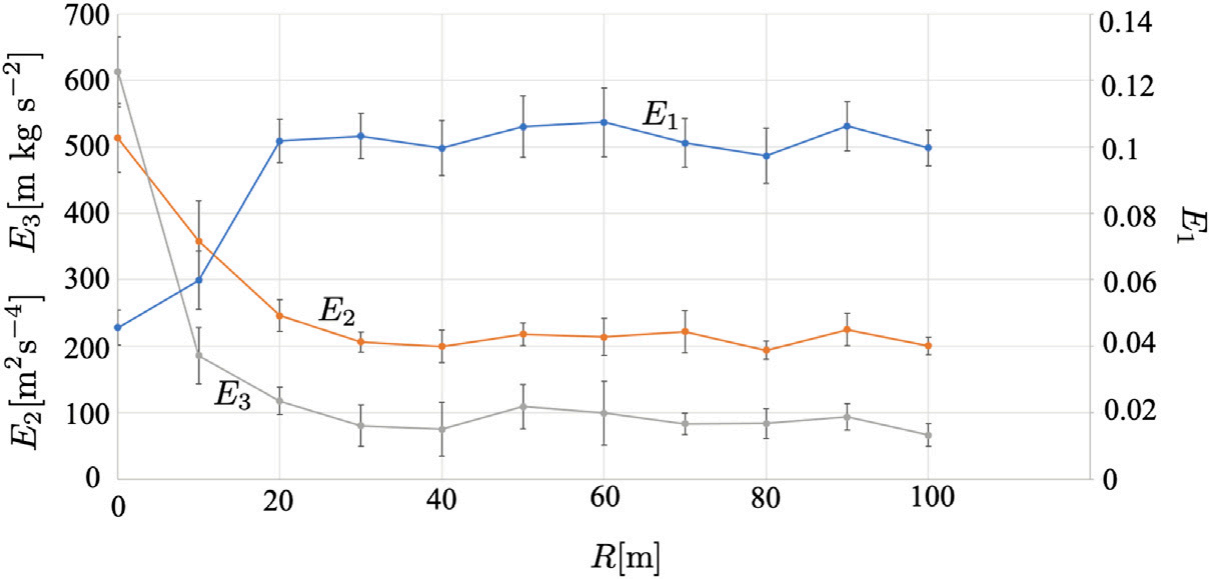
Result when the radius of the observation range *R* is varied. The average over five trials is shown. The error bars denote the standard deviation.

Figure 10 shows the results when the radius of the observation range *R* is changed. Supplementary Movies S3, S4 show the cases with *R* = 10 and 80 m, respectively. We find that *E*_1_ increases, whereas *E*_2_ and *E*_3_ decrease as *R* increases. For a small *R*, agents cannot find or avoid other agents until they are in proximity to them. Thus, each agent tends to move straight at a high speed until it finds the other agents. When it finds nearby agents with a high risk of collision, it abruptly attempts to avoid other agents but sometimes collides with them.

**FIGURE 11:**
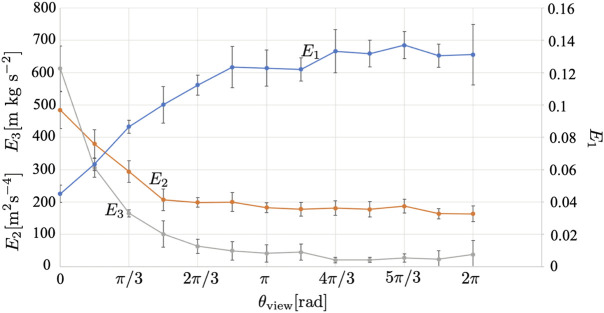
Result when the central angle of the observation range *θ*_view_ is varied. The average over five trials is shown. The error bars denote the standard deviation.

Figure 11 shows the results when the central angle *θ*_view_ of the observation range was changed. Supplementary Movies S5, S6 show the cases where *θ*_view_ = *π*/6 and 3*π*/2, respectively. We found that *E*_1_ increased, whereas *E*_2_ and *E*_3_ decreased as *θ*_view_ increased. It is difficult to identify and avoid agents with a high risk of collision for a small *θ*_view_. Thus, the dependence of *θ*_view_ was similar to that of *R*.

**FIGURE 12:**
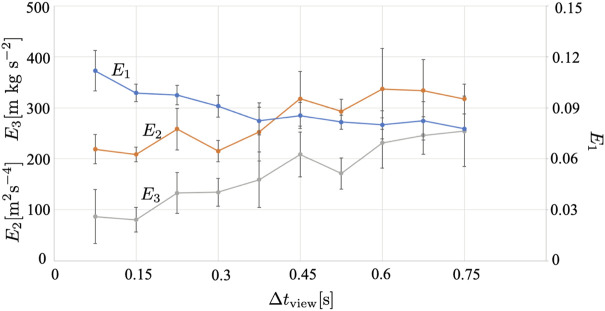
Result when the time interval for the update of the fan-shaped observation range Δ*t*_view_ is varied. The average over five trials is shown. The error bars denote the standard deviation.

Figure 12 shows the results when the time interval for updating the fan-shaped observation range, Δ*t*_view_ was changed. Supplementary Movies S7, S8 show the cases where Δ*t*_view_ = 0.225 s and 0.675 s, respectively. We found that *E*_1_ did not depend considerably on Δ*t*_view_, whereas *E*_2_ and *E*_3_ increased as Δ*t*_view_ increased. For a large Δ*t*_view_, it is difficult to find and avoid other agents; thus, each agent sometimes needs to avoid other agents abruptly but fails to avoid them, and they collide with each other.

**FIGURE 13:**
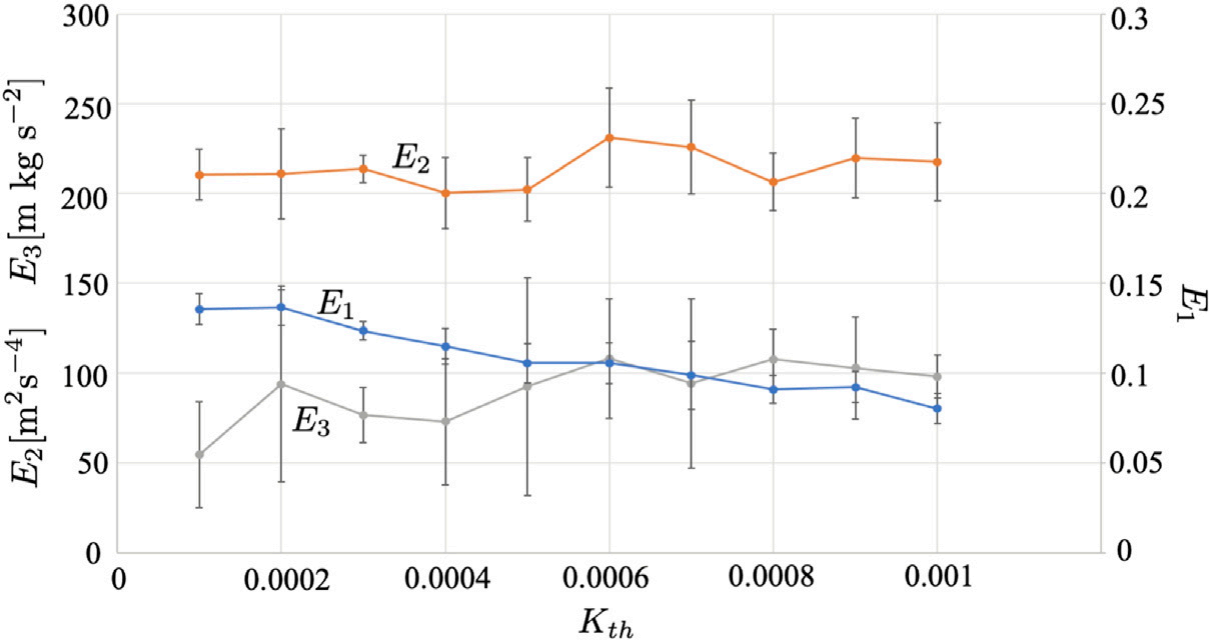
Result when the threshold of collision risk *K*_*th*_ is varied. The average over five trials is shown. The error bars denote the standard deviation.

Figure 13 shows the results when the threshold of the collision risk level *K*_*th*_ is changed. Supplementary Movies S9, S10 show the cases in which *K*_*th*_ = 0.0001 and 0.001, respectively. Although *E*_2_ does not depend considerably on *K*_*th*_, *E*_1_ decreases, and *E*_3_ increases as *K*_*th*_ increases. For a small *K*_*th*_ (Supplementary Movie S9), the agents tend to focus on many nearby agents; thus, they can avoid collisions when speed is lost. In contrast, for a large *K*_*th*_ (Supplementary Movie S10), the agents focus only on a few nearby agents; thus, they can move at high speeds, although collisions cannot be avoided. Moreover, as *K*_*th*_ increases, the sensing and calculation costs decrease because many nearby agents are ignored.

### 4.3 Comparison with the previous model (Kano et al., 2021)

**FIGURE 14:**
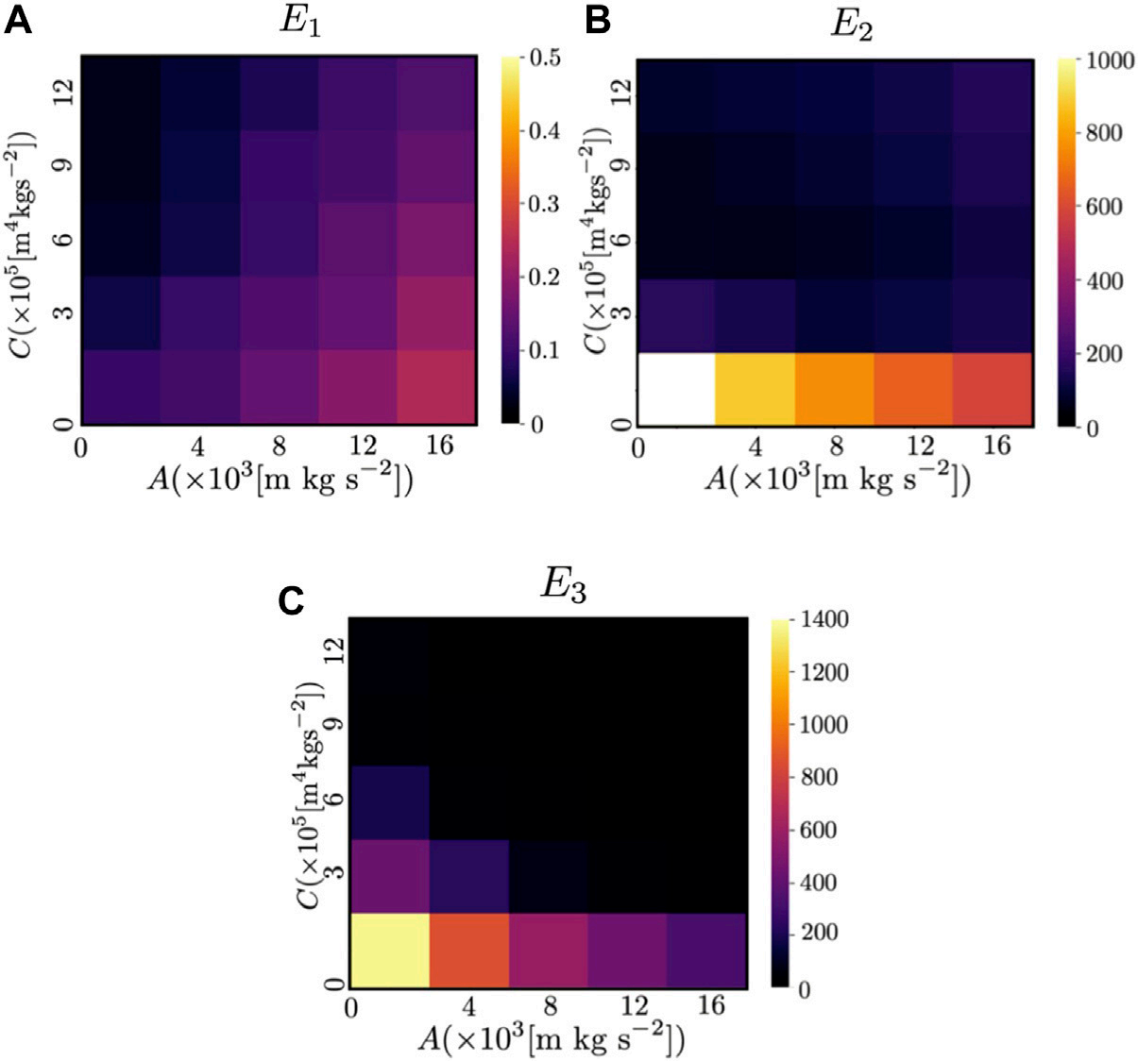
Color map of **(A)** *E*_1_, **(B)** *E*_2_, and **(C)** *E*_3_ in the previous study when *N* = 50. The color indicates the values of the indices (*E*_1_, *E*_2_ and *E*_3_) when the parameters *A* and *C*, which determine the magnitude of the repulsive force from other agents and the avoidance force based on the prediction of the future motion of other agents, respectively [see Eq. 5 in (Kano et al., 2021)], were varied. The white areas indicate that the value exceeded the range shown in the color map.

**FIGURE 15:**
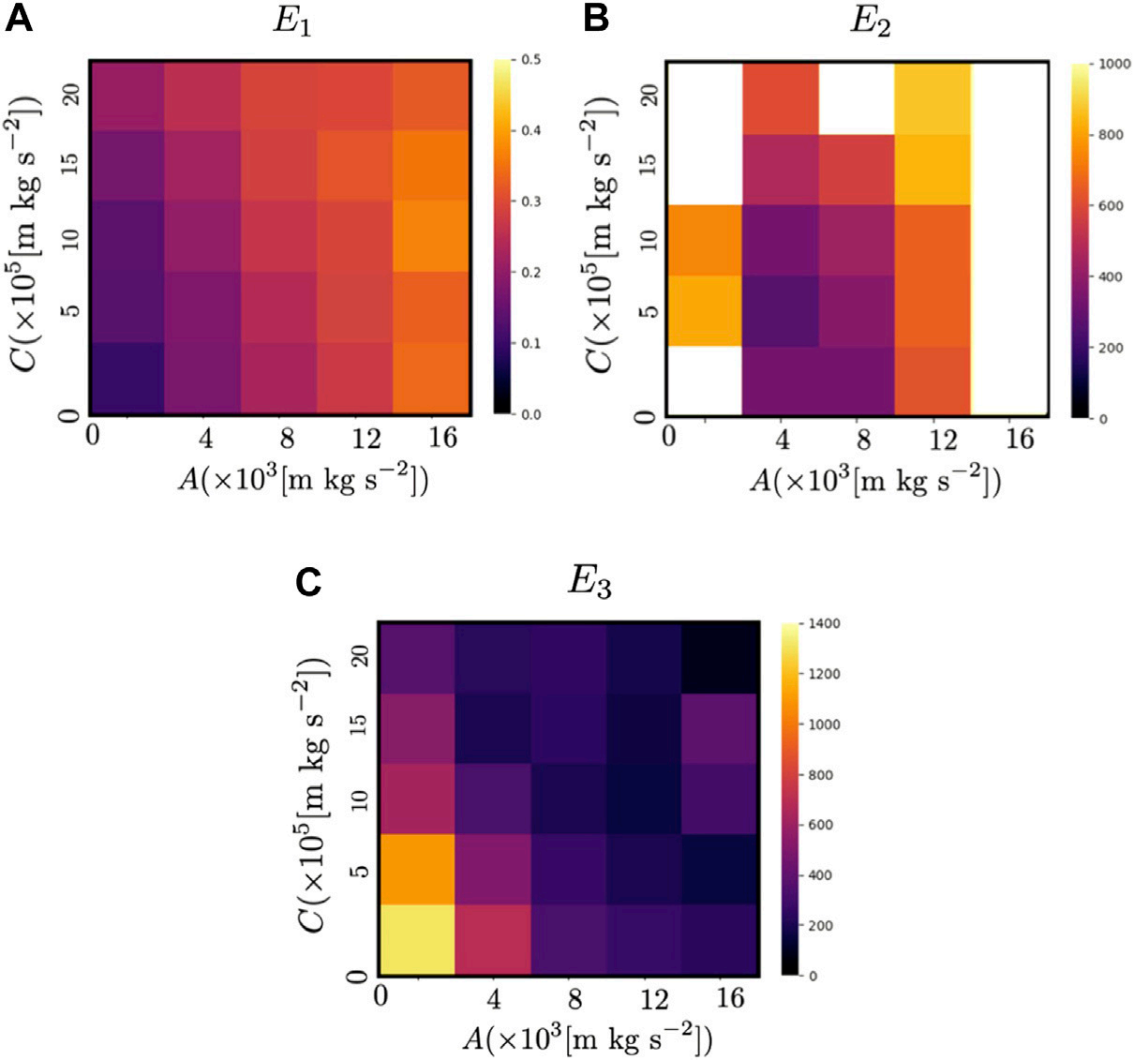
Color map of **(A)** *E*_1_, **(B)** *E*_2_, and **(C)** *E*_3_ for the proposed model when *N* = 50. The color indicates the values of the indices (*E*_1_, *E*_2_ and *E*_3_) when the parameters *A* and *C*, which determine the magnitude of the repulsive force from other agents and the avoidance force based on the prediction of the future motion of other agents, respectively (Eqs. 12, 16) were varied. White areas indicate that the value exceeded the range shown in the color map.

The performance of the proposed model was compared with that of a previous model (Kano et al., 2021). In this experiment, *N* = 50, *θ*_view_ = *π*, Δ*t*_view_ = 0.375 s, and the parameters *A* and *C*, which determine the magnitude of the repulsive force from other agents and the avoidance force based on the prediction of the future motion of other agents, respectively, were varied. The results of the previous and proposed models are shown in Figure 14 and Figure 15, respectively. Because the sensing and calculation costs of the proposed model are considerably lower than those of the previous model (Kano et al., 2021), the performance of the proposed model is inevitably worse than that of the previous model. However, when *A* = 4.00 × 10^3^[m kg s^−2^] and *C* = 5.00 × 10^5^[m kg s^−2^], *E*_1_ and *E*_2_ of the proposed model are approximately 1.9 times and *E*_3_ of the proposed model is 2.3 times larger than that of the previous model. Thus, when *A* and *C* are properly chosen, the deterioration in performance is not severe, even though the sensing and calculation costs are considerably reduced.

## 5 Discussion

We proposed a decentralized control scheme for mobile agents that enables quick, smooth, and safe movement with limited sensing capability of each agent by introducing active sensing. To the best of our knowledge, this is the first study in which a control scheme based on active sensing was designed to achieve smooth collision avoidance in multi-robot systems. Through simulations, we confirmed that the proposed model performed reasonably well. Moreover, when the parameters were selected appropriately, the performance deterioration compared with that observed in a previous study (Kano et al., 2021) was not as severe, even though the amount of sensing was considerably reduced. Because the calculation cost for each agent is low, we believe that the proposed model can be used for various applications, such as those involving robots in warehouses and drones.

An advantage of the proposed control scheme is that its calculation cost is considerably lower than that of our previous scheme (Kano et al., 2021). Although deriving the decrease in the calculation cost is difficult, we can approximately estimate that the number of agents on which each agent focuses is approximately or less than *θ*_view_/(2*π*). Furthermore, because each agent focuses only on agents whose collision risk is above threshold *K*_*th*_, the calculation cost decreases as *K*_*th*_ increases. Another advantage of the proposed control scheme is that each agent does not need to know the directions in which nearby agents intend to move, (*i.e.,* the unit vector **e**_*i*_ of the nearby agent *i*). In our previous control scheme, however, it was necessary for each agent to know this information.

However, several problems remain unresolved.

• Limitation of the control scheme: We found that the proposed control algorithm did not perform well when the density of the agents was large. This is likely because the prediction of the agents’ motions was oversimplified. We intend to further improve the control scheme such that its performance is improved without increasing the calculation cost.
• Technical problem: It is technically difficult to instantly change the direction of the fan-shaped observation range because mobile robots usually have a lidar sensor that is statically linked to the robot. Thus, the proposed model remains somewhat unrealistic. These problems should be addressed in future hardware realizations.
• Further extensions: We intend to extend the proposed control scheme to the three-dimensional case and the case where obstacles exist.

## Data Availability

The original contributions presented in the study are included in the article/Supplementary Material, further inquiries can be directed to the corresponding author.

## Supplementary material

Supplementary Movie S1 Simulation when N = 2.

Supplementary Movie S2 Simulation when N = 20

Supplementary Movie S3 Simulation when R = 10.

Supplementary Movie S4 Simulation when R = 80

Supplementary Movie S5 Simulation when θ_view_ = π/6.

Supplementary Movie S6 Simulation when θ_view_ = 3π/2.

Supplementary Movie S7 Simulation when Δt_view_ = 0.225 s.

Supplementary Movie S8 Simulation when Δt_view_ = 0.675 s.

Supplementary Movie S9 Simulation when K_th_ = 0.0001.

Supplementary Movie S10 Simulation when K_th_ = 0.001.
